# A hepatocyte targeted corn peptide—galactooligosaccharide nanoparticle for the alleviation of ethanol-induced damage in cells

**DOI:** 10.3389/fnut.2026.1987391

**Published:** 2026-10-27

**Authors:** Mingyi Zhou, Xiying Jiang, Chen Li, Yuxiao Wang, Sijia Hao

**Affiliations:** 1 College of Food Science and Engineering, Jinzhou Medical University, Jinzhou, China; 2 College of Food Science and Engineering, Bohai University, Jinzhou, China; 3 State Key Laboratory of Marine Food Processing and Safety Control, Dalian Polytechnic University, Dalian, Liaoning, China; 4 National Engineering Research Center of Seafood, Dalian Polytechnic University, Dalian, Liaoning, China

**Keywords:** alcohol-induced liver damage, corn peptide, galactooligosaccharides, hepatocyte-targeted, Maillard reaction

## Abstract

Alcoholic liver disease is defined as damage of liver structure and function caused by prolonged heavy alcohol consumption, and novel preventive and treatment options are needed. Corn peptide (CP), which is produced through enzymatic hydrolysis of corn protein, is a bioactive polypeptide that has been used for hangover relief and liver support. To alleviate alcohol-induced damage to liver cells, we designed hepatocyte-targeted nanoparticles consisting of CPs attached via the Maillard reaction to galactooligosaccharides (GOS). The resulting CPs-GOS measured 73.19 nm in diameter, presented a uniform spherical morphology, and exhibited e storage stability, free radical scavenging ability, and biocompatibility. Importantly, the high stability and the efficient accumulation of CPs-GOS in LO2 cells can be attributed to the galactosyl component of the GOS. In cultured hepatocytes, treatment with CP-GOS increased ethanol-induced cell viability from 48.87 to 75.18%, and reduced oxidative stress. Our study demonstrated great potential for the CPs-GOS nanoparticle system as an intervention for alcoholic liver disease.

## 1 Introduction

Alcoholic liver disease (ALD) is a chronic disease resulting from prolonged and heavy alcohol consumption. And the disease can progress from fatty liver to liver fibrosis, and cirrhosis and can eventually lead to death (1). While the precise pathogenesis of acute ALD has not been fully clarified, current evidence indicates that alcohol metabolism induces oxidative stress in hepatocytes, leading to the production of acetaldehyde, inflammatory responses, and cellular injury (2). In cases of severe alco-holism, extensive hepatocyte necrosis may occur, potentially progressing to liver failure (3). The clinical manifestations of acute alcoholic liver injury are often nonspecific and can include nausea, malaise, and low-grade fever (4). The progression of ALD typically begins with an often-asymptomatic stage of hepatic steatosis (alcoholic fatty liver). This can advance to alcoholic hepatitis, characterized by overt inflammation (5, 6). Without intervention, the disease may continue to progress to alcoholic liver fibrosis and, ultimately, cirrhosis, which is often irreversible. So, ALD is among the predominant types of chronic hepatic diseases in the world (7, 8).

Bioactive compounds isolated from natural plants have multiple health-promoting effects, particularly in preventing and mitigating pathophysiological processes associated with alcohol metabolism (9). Several classes of bioactive compounds have demonstrated efficacy in ameliorating alcohol-induced liver injury. Representative protective agents include polyphenols (e.g., curcumin), specific proteins and peptides (e.g., corn peptides (CPs)), polysaccharides (e.g., Ganoderma lucidum polysaccharides), and micronutrients (e.g., vitamin E) (10). CPs are small bioactive polypeptides obtained from enzymatically or fermentatively hydrolyzed corn protein meal; in 2010, they were authorized as a new food resource in China (11, 12). Apart from protecting against alcoholic liver injury, these peptides possess multiple bioactivities such as antioxidant, blood pressure-lowering, blood glucose-regulating, and lipid-reducing properties, collectively supporting overall health (13, 14). The mechanisms by which CPs promote health have been demonstrated to be mediated in large part by their abundant leucine and alanine residues.

Despite its therapeutic potential, the efficacy of CPs is limited by their poor acid stability and susceptibility to enzymatic hydrolysis, leading to rapid systemic clearance, a short half-life, and consequently low oral bioavailability. Various formulation approaches have been explored to address these shortcomings (15, 16). Jiao et al. (17) re-ported a high-efficiency strategy for preparing nanoparticles from peptides derived from the corn protein zein; these nanoparticles improved the stability of lutein under conditions mimicking the gastrointestinal environment. Seyed et al. (18) utilized spray-drying with maltodextrin and β-cyclodextrin as wall materials to obtain powders of corn flower pollen peptides with improved thermal stability. Despite these advances, the therapeutic application of CPs still faces a series of challenges related to stability and targeting efficiency. Specifically, the targeted delivery of peptides to the liver would be desirable in order to enhance local concentrations of therapeutic agents (19). Consequently, the construction of active targeting strategies to improve the bioavailability of CPs would be likely to have positive impacts on ALD patient outcomes.

The Maillard reaction refers to a cascade of non-enzymatic reactions initiated between the amino groups of proteins and carbonyl groups of reducing sugars (20). As a naturally occurring and food-grade process, it proceeds without requiring extraneous chemical catalysts. The protein-sugar conjugates formed via this reaction exhibit enhanced functional properties, including improved solubility, stability, emulsifying capacity, and antioxidant activity (21). In prior research, we engineered a hepatocyte-targeted delivery system by combining sea cucumber peptide and galactose through the Maillard reaction, and we showed that this formulation improved the water solubility and bioavailability of silymarin (22). These accomplishments have stressed the potential utility of conjugates formed through the Maillard reaction for advancing the field of delivery systems, particularly for nutraceuticals and bioactive compounds (23).

Liver-targeted delivery systems typically prolong the retention time of active compounds in the organ by taking advantage of specific receptor-ligand interactions (24). The asialoglycoprotein receptor (ASGPR) is found on the surface of hepatic parenchymal cells. As this receptor specifically recognizes galactose, galactosylated ligands have been shown to be specifically bound by ASGPR and thus to be targeted to the liver (25). Based on this rationale, the Maillard reaction to glycosylate CPs and form CPs-GOS conjugates in the absence of any additional chemical reagents. We characterized the synthesis of the conjugate and then assessed its morphological and physicochemical properties. Its antioxidant capacity was determined through free radical scavenging assays. We performed preliminary *in vitro* analyses of the safety profile of the nanoparticle and evaluated its protective effects against alcohol-induced hepato-cellular injury.

## 2 Materials and methods

### 2.1 Materials

CPs were purchased from Shandong Baixing Biotechnology Co Ltd. (Shandong, China). GOS was procured from Shanghai McLean Biochemical Technology Co. Ltd. (Shanghai, China). DMEM medium was procured from Gibco (USA). Hoechst 33342, DCFH-DA, and FITC were procured from Shanghai Biyuntian BioTechnology Co (Shanghai, China).

### 2.2 Synthesis of CPs-GOS nanoparticles

CPs-GOS were prepared by the wet-Maillard reaction. CPs and GOS were dispersed in 10 mL of ultrapure water at ratios of 1:1, 1:2, 2:1, or 4:1. The mixed liquor was stirred well at 25 °C and then incubated at 80 °C for 0, 2, 4, 6, or 8 h. The Maillard reaction was halted by placing the CPs-GOS in ice water for 10 min. Subsequently, the sample underwent freeze-drying and was kept in the dark.

### 2.3 Determination of grafting degree

The content of free amino groups was assessed using the o-phthalaldehyde (OPA) protocol as mentioned previously (26). The OPA reagent was prepared by dissolving 0.04 g of OPA in 1 mL of methanol, with the subsequent addition of 2.5 mL of 20% (w/v) SDS, 25 mL of 0.1 mol/L sodium tetraborate, and 100 μL of β-mercaptoethanol. A portion (100 μL) of a CPs-GOS solution (10 mg/mL) was added with 2 mL of OPA and reacted at 35 °C for 2 min. The absorbance at 340 nm was detected rapidly with a UV–visible spectrophotometer (Shimadzu, Japan). The degree of glycation (DG) was computed as the evaluation index according to Equation (1), where ODB and ODA are the absorbances before and after reaction, respectively.


$$\mathrm{DG}(\%)=\frac{OD_{B}-OD_{A}}{OD_{B}}\times 100$$ (1)

### 2.4 Determination of browning index

The degree of the Maillard reaction was assessed quantitatively by determining the browning index (BI). Specifically, the BI values of covalent conjugates prepared with different reaction times was estimated by measuring absorbance at 294 nm (OD294) and 420 nm (OD420) (27).

### 2.5 Fluorescence spectroscopy

Fluorescence spectra were measured using a fluorescence spectrophotometer (Hitachi, Japan). The slit width was 5 nm, the excitation wavelength was 280 nm, the emission wavelength range was 300 to 450 nm.

### 2.6 Determination of zeta potential

Solutions of samples of CPs-GOS produced with different glycosylation times were prepared at concentrations of 0.05 g/mL. One milliliter of the solution to be tested was dispensed into the given pool and then analyzed with the Zeta potential analyzer (Malvern Instruments, UK). Each sample was detected in parallel five times, and the measured results were averaged.

### 2.7 Structural analysis

The chemical structures of CPs, GOS, and CPs-GOS were analyzed using a Fourier transform infrared (FTIR) spectrometer (Shimadzu, Japan) over the wavenumber region of 4,000 to 400 cm^−1^. Crystalline morphologies of various nanoparticles were investigated by X-ray diffractometry (XRD) (Rigaku, Japan). XRD was performed with a dwell time of 0.1 s and 2θ = 5–50°.

### 2.8 Morphological analysis

To perform atomic force microscopy (AFM), samples of CP-GOS were dropped onto a mica substrate. When the samples were completely dry, microscopic images were collected using an AFM system (Digital Instruments Inc., USA). For transmission electron microscopy (TEM), a diluted sample (7 μL) was applied to a copper grid and dried at ambient temperature and then stained with phosphotungstic acid. TEM photos were shot using a microscope from JEOL Ltd. (Japan).

### 2.9 Determination of stability

Differential scanning calorimetry (DSC) was enforced with a thermal analyzer (TA Q2000) over a range of 30 °C to 180 °C. For visual inspection of stability, freshly prepared CPs-GOS conjugates were stored at 4 °C, and photographic documentation was performed every 7 days for 4 weeks to facilitate the monitoring of color changes.

### 2.10 *In vitro* assessments of antioxidant activity

Antioxidant activity was quantified with a DPPH-based assay according to the method reported by Alabdullah et al. (28). Twenty microliters of sample solution or blank solution were added to 2 mL of 0.1 mol/L DPPH dissolved in 95% ethanol. After incubation for 0.5 h at 25 °C in the dark, the absorbance values (λ = 517 nm) of the supernatants of the blank reaction (OD_blank_) and the sample reaction (OD_sample_) were determined. The free radical scavenging activity was computed using the Equation (2).


$$\text{DPPH scavenging activity}\;(\%)=(1-\frac{OD_{\text{sample}}}{OD_{\text{blank}}})\times 100$$ (2)

The ABTS radical scavenging method was quantified essentially as reported (29). The mixed liquor containing 4 mL ABTS working solution and either 1 mL of a solution containing CP-GOS was incubated for 5 min at 25 °C, and then the absorbance was detected at 734 nm. The ABTS radical scavenging activity was computed using Equation (3).


$$\text{ABTS clearance activity}\;(\%)=\frac{OD_{\text{control}}-OD_{\text{sample}}}{OD_{\text{control}}}\times 100$$ (3)

Hydroxyl radical clearance was determined using the procedure described by Ma et al. (30). A 0.5 mL blank sample or sample containing CPs of CPs-GOS was added with 0.5 mL of 6 mmol/L FeSO_4_ and 0.5 mL of 6 mmol/L H_2_O_2_. The mixed liquor was incubated at 25 °C for 10 min, and then 0.5 mL of 6 mmol/L salicylic acid in ethanol was mixed and incubated at 37 °C for 0.5 h. The absorbance value at 510 nm, and the hydroxyl radical clearance activity was computed following the Equation (4).


$$\begin{array}{l} \text{Hydroxyl radical clearance activity}\;(\%)\\ =(1-\frac{OD_{\text{sample}}-OD_{\text{control}}}{OD_{H2o2}-OD_{\text{control}}})\times 100 \end{array}$$ (4)

The reducing capacity of the glycosylated products was estimated by the ferric chloride reduction method (31). A portion (0.5 mL) of a CPs-GOS solution was added sequentially with 2.5 mL of 0.2 mol/L phosphate buffer (pH 6.6), 2.5 mL of 1% (w/v) potassium ferricyanide solution, and then 2.5 mL of 10% trichloroacetic acid. After incubation at 50 °C for 20 min, the mixed liquor was cooled at 20 °C, and the reaction was terminated by centrifugation at 3000 r/min for 20 min. One milliliter of the supernatant was added to 1.0 mL of ultrapure water and 20 μL of 0.1% ferric chloride. This reaction was reacted for 15 min with constant mixing. The absorbance value was at 700 nm was taken as a measure of reducing capacity.

### 2.11 Cytotoxicity assay

The cytotoxic of CPs-GOS produced with different reaction times were determined by the MTT assay. LO2 cells in the logarithmic growth stage were put into 96-well plates at a number of 1 × 10^5^ cells per well, and were reacted overnight in a CO_2_ incubator. CPs-GOS samples (160 μg/mL) were dissolved in cell growth medium and added to the cells, which were reacted for 12 h. cell survival rate was quantified using a standard MTT assay kit as described and the Calcein AM/PI dye assay as previously described by literature (32). An ethanol injury model was produced in LO2 cells and manipulated as previously described by our group (CPs-GOS samples (low: 40 μg/mL, medium: 80 μg/mL, high: 160 μg/mL, alcohol: 0.8 mol/mL, time: 12 h)) (22).

### 2.12 Cellular uptake assay

LO2 cells were added in 6-well cell plates and incubated for 1 day. DMEM medium containing FITC@CPs-GOS was added, next the cells were reacted for more 2 or 4 h. Cellular nuclei were labeled with Hoechst 33342 (10 μL) for 10 min. Cellular uptake was observed with an inverted fluorescence microscope, and the rate of nanoparticles was measured by flow cytometry.

### 2.13 Reactive oxygen species (ROS) measurements

LO2 cells were put in 12-well plates and reacted for 24 h. The cells were then treated with 0.8 mol/L ethanol for 12 h. After removal of the ethanol, cells were mixed with low, middle, and high concentrations of nanoparticles for 12 h. The treated cells were labeled with DCFH-DA for 0.5 h and then observed with a fluorescence inverted microscope.

### 2.14 Hemolysis assays

The commercially available defibrinated sheep blood was put into a tube and centrifuged at 5000 r/min for 15 min. Red blood cell suspensions (2%) were created by adding PBS (negative control, 0% hemolysis), water (positive control, 100% hemolysis), or PBS containing CP-GOS (0.25, 0.5, 1, 1.5, or 2 mg/mL). The suspensions were reacted at 37 °C for 1 h or 3 h and then centrifuged at 5000 r/min for 15 min. The samples were photographed, and the absorbance values of the supernatants were detected at 540 nm. The hemolysis activities were computed according to Equation (5).


$$\text{Hemolysis activity}\;(\%)=\frac{OD_{\text{sample}}-OD_{\text{negative}}}{OD_{\text{positive}}-OD_{\text{negative}}}\times 100$$ (5)

### 2.15 Statistical analysis

Each experiment was performed with no fewer than three replicates and the quantitative findings are displayed as mean ± SD. Figures were prepared with Origin Pro 2022. The symbols # and * are used to represent significant differences between groups, with significance defined as *p* < 0.05.

## 3 Results and discussion

### 3.1 Preparation and characterization of CPs-GOS nanoparticles

CPs-GOS nanoparticles were constructed by utilizing the wet Maillard reaction (Figure 1A). Figure 1B shows the changes in the color of CP and GOS when incubated in different ratios and for different times. With increased CP content or increased heat-up time, the hue of the resulting CPs-GOS shifted from yellow to a darker orange-brown color. In order to measure the degree of the Maillard reaction, we deter-mined the amount of free amino groups remaining and thus the DG (33). As illustrated in Figure 1C, the highest DG (36.48%) was obtained when the conditions included glycosylation time of 8 h and a ratio of CP to GOS of 4:1.

**Figure 1:**
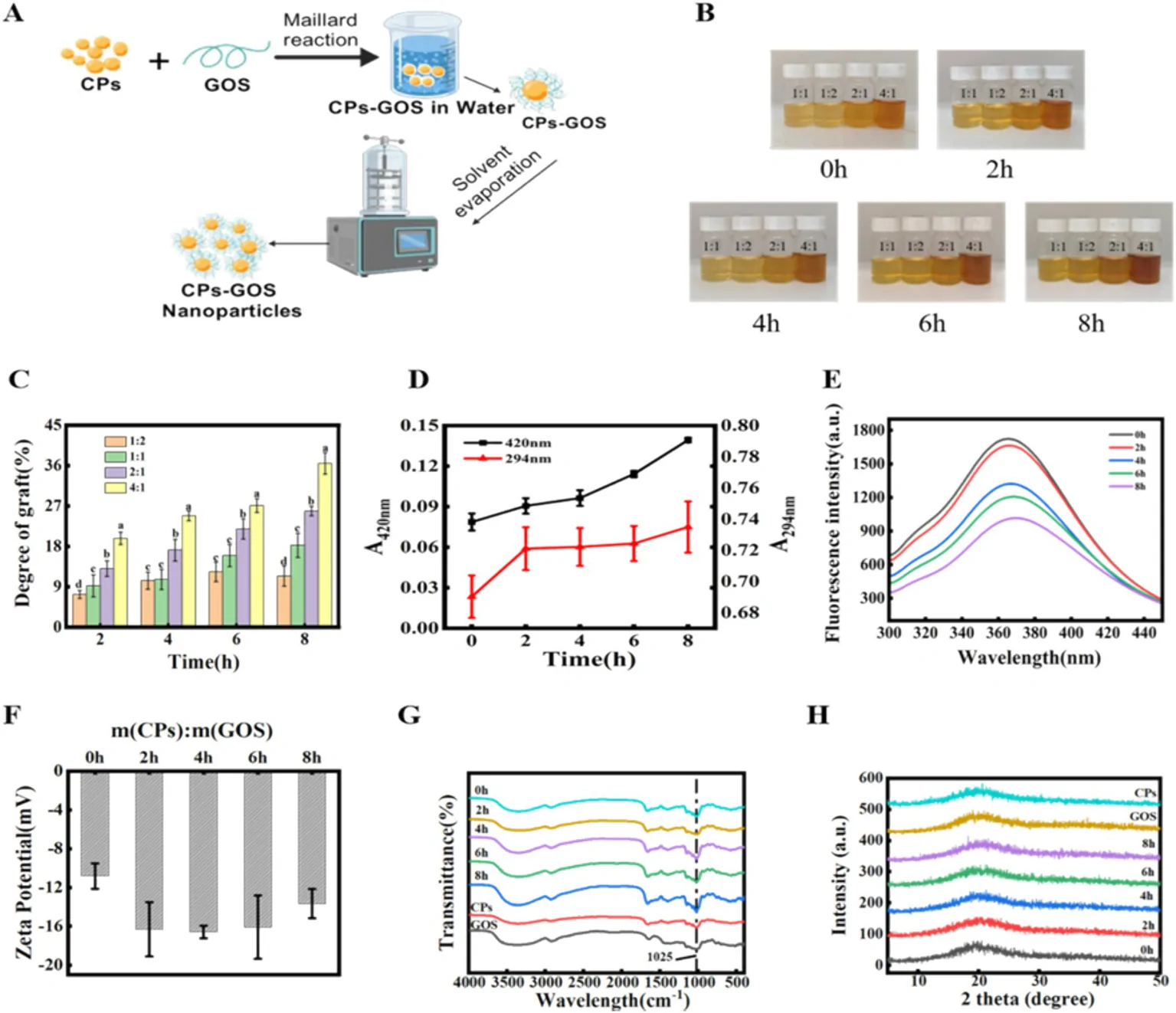
Synthesis and initial characterization of CP-GOS nanoparticles. **(A)** Schematic diagram of the design of CPs-GOS nanoparticles and of the synthesis process. **(B)** Representative images of reaction mixtures with different reagent mass ratios (CPs: GOS) and incubated for different times. **(C)** Degree of graft was measured by determining free amine groups. Letters indicate significant differences (*p* < 0.05). **(D)** Absorbances of solutions containing equivalent amounts of product from reactions of different times (bars: 294 nm, scatter: 420 nm). **(E)** Fluorescence spectra. **(F)** Zeta potentials. **(G)** FTIR spectra. (H) XRD spectra.

In its early stages, Maillard reactions are known to be characterized by ketones and aldehydes that exhibit maximum absorbances in the near-UV range (294 nm); as the reaction proceeds, nitrogen-containing brown pigmented substances are made, which means that later stages are characterized by increased absorbance at 420 nm (34). In our synthesis reactions, the absorbance values at both 294 and 420 nm increased over time (Figure 1D), consistent with the production of intermediate and end products of the Maillard reaction. Notably, the Maillard reaction can also produce fluorescent sub-stances that have been used to analyze the DG; however, we observed a time-dependent decrease in fluorescence intensity (Figure 1E). The Maillard reaction resulted in a masking of the intrinsic fluorescence of CPs, consistent with earlier findings (35, 36).

During the course of the reaction, the absolute value of the Zeta potential of CPS-GOS was initially elevated and then diminished (Figure 1F). The initial increase is consistent with the electrostatic interaction between CP and GOS during the recombination process (37). At later stages of the reaction (6–8 h), however, the covalent com-plex produced in the last stage of the Maillard reaction led to a shielding of the surface of the CP, which would be expected to increase the number of charged residues due to the ionization of polar groups and cause the potential to decrease. Therefore, these results demonstrated that the Maillard reaction was successful and that CPs were bound to GOS.

FTIR spectroscopy is a common tool for investigating the interactions between proteins and polysaccharides and the properties of conjugates formed by the Maillard reaction (38). When we analyzed CP by FTIR (Figure 1G), a wide absorption band was observed in the spectrum at 3700–3000 cm^−1^, which was attributed to O-H/N-H stretching vibrations. Bands corresponding with -CH_3_- and -CH_2_- were observed at 2990–2850 cm^−1^, these bands were mainly due to the stretching vibrations of C-H. A pronounced absorption peak was observed at 1671 cm^−1^, which is a distinctive absorption peak of C=O stretching vibrations, indicating that the sample contained a uronic acid structure. The peak at 1025 cm^−1^ may be attributed to C-O tensile vibration. In the GOS FTIR spectrum, bands at 3375, 2917, 1,651, 1,159, and 1,016 cm^−1^ were attributed to O-H stretching vibrations, C-H stretching vibrations, -C=C- stretching vibrations, X-H in-plane bending vibrations, and X-Y stretching vibration, respectively. After the coupling reaction to form CPs-GOS, the amide I band and the C-O-C antisymmetric stretching band moved to 1,672 and 1,028 cm^−1^, respectively, indicating that a Schiff base and glycosylamine were formed initially in the Maillard reaction. The two absorption peaks were enhanced by the C-O bond and the stretching vibration of the hydroxyl group, which allowed grafting of anionic GOS onto CPs. These findings demonstrated that nanoparticles consisting of CP and GOS were formed by the Maillard reaction and that the materials were successfully joined by the glycosylation reaction (39).

We next used XRD to detect the impact of reaction time on the structures of the CPs-GOS nanoparticles. The XRD patterns of the grafts of CPs, GOS, and CPs-GOS are shown in Figure 1H. The maximum values of 2θ of the covalent grafts generated with 0, 2, 4, 6, and 8 h of reaction were 19.14°, 19.4°, 18.64°, 19.16°, and 18.28°, respectively. The grafts produced with different reaction times all showed a wide peak around 2θ = 19°, which may be attributed to the development of an amorphous carbon structure inside the molecule, and it is consistent with results from several previous reports (40). Taken together, these data provide further evidence that CPs can be coupled to GOS via the Maillard reaction.

### 3.2 Morphological analyses of CPs-GOS nanoparticles

The morphologies and surface characteristics of the nanoparticles were characterized using AFM and TEM. As illustrated in Figure 2A, the AFM analysis demonstrated that the nanoparticles possessed a uniform spherical structure. TEM images (Figure 2B) provided further confirmation that the nanoparticles exhibited a smooth spherical shape. According to an ImageJ-based analysis of the microscopic images, the particles ranged in diameter from approximately 50 to 100 nm, with the average diameter being 73.19 ± 1.4 nm.

**Figure 2:**
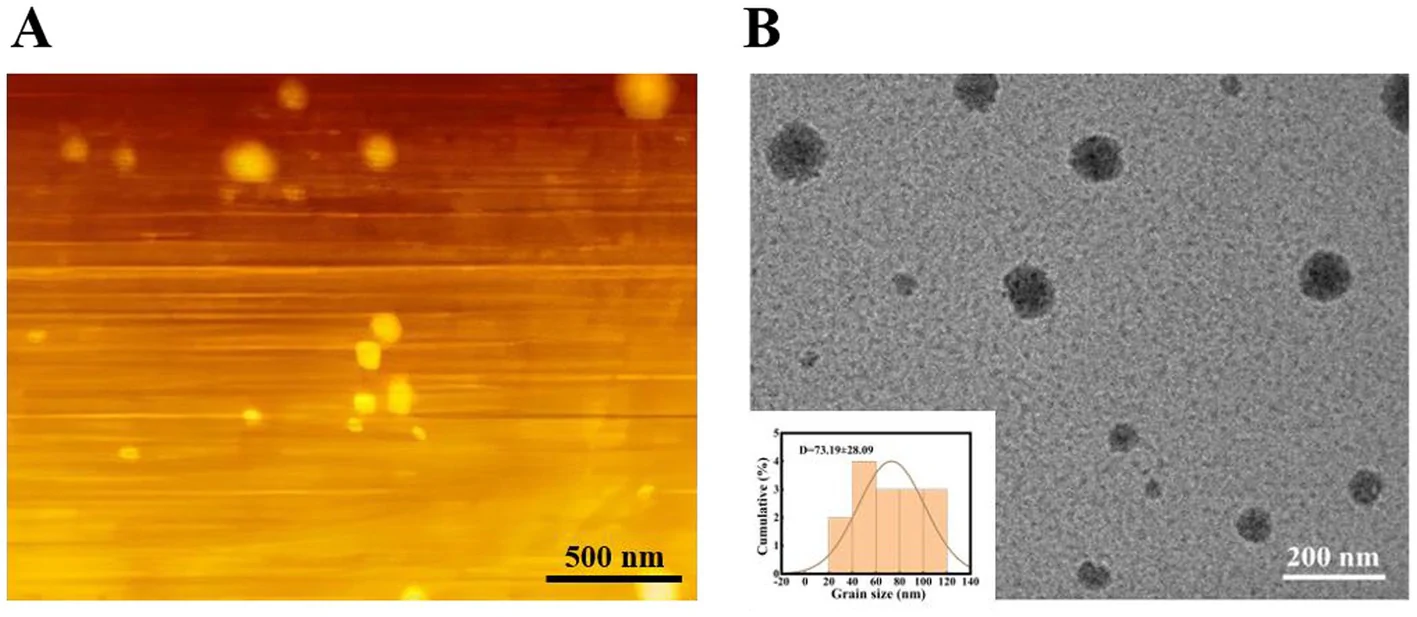
Structural characterization of CPs-GOS nanoparticles. **(A)** Representative AFM image. **(B)** Representative TEM image. Inset: Particle size distribution as determined by TEM.

### 3.3 Analysis of the stability of the CPs-GOS nanoparticles

As illustrated in Figure 3A, over 28 days of storage of mixtures containing either the pure peptide or CPs-GOS at 4 °C in the dark, we observed no visible changes, such as precipitation, aggregation, or discoloration. These results suggest that the grafted conjugates possess robust structural stability under the tested conditions.

**Figure 3:**
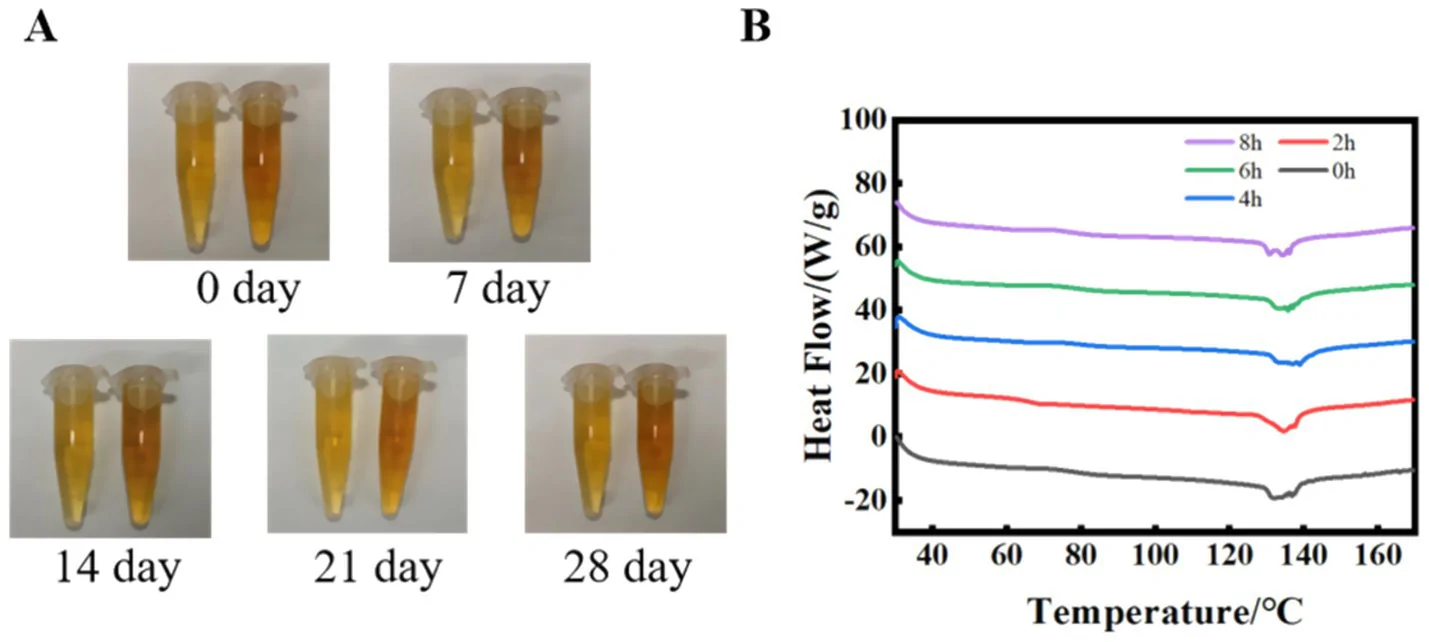
Stability analyses. **(A)** Representative images of pure CP (left) and CPs-GOS (right) stored at 4 °C for the noted times. **(B)** Thermal stability of CPs-GOS nanoparticles produced with different reaction times, as determined by DSC.

As illustrated in Figures 3B, a DSC analysis revealed differences in the melting temperatures of CPs-GOS formed with different reaction times. The initial increase of heat flow followed by a decrease suggested the establishment of a stable network structure through hydrogen bonding and electrostatic and hydrophobic interactions between CPs and GOS, thereby enhancing the thermal stability of the nanoparticles.

### 3.4 CPs-GOS exhibits robust antioxidant activity

The antioxidant activity of CPs-GOS nanoparticles was evaluated by four independent assays, in which we measured DPPH scavenging activity, ABTS+ free radical scavenging activity, OH- free radical scavenging activity, and reducing power. As il-lustrated in Figure 4, these methods yielded similar results, thus demonstrating the strong antioxidant activity of the formulation. Specifically, as illustrated in Figure 4A, according to the DPPH assay, the mean free radical scavenging activity of pure CP was 24.51%, while the mean activity of the CPs-GOS conjugate increased to 76.19% after 8 h of reaction. The reaction time-dependent increase of activity of CPs-GOS and the higher activity of CPs-GOS relative to that of CP are consistent with a model in which the formation of covalent bonds with GOS reduces intermolecular hydrogen bonding of CP, thus increasing the free radical scavenging ability. In the ABTS+ assay (Figure 4B), the mean free radical scavenging activity of pure CP was 26.22%, while the mean free radical scavenging activity of CP-GOS increased to 72.61% after 8 h of reaction. These increases likely reflect the production of pyrrole, thiophene, or other specific reactive structures during the Maillard reaction. These aromatic compounds can increase anti-oxidant capacity by promoting electrophilic addition reactions (41). In Figure 4C, the OH- free radical scavenging abilities of the CPs-GOS mixture also increased with time of reaction. The mean free radical scavenging activity of the CPs-GOS mixture in-creased from 26.88% at the start of the reaction to 72.69% after 8 h of reaction. We also employed the potassium ferrocyanide reduction method, in which the reduction of potassium ferricyanide (K₃[Fe(CN)₆]) to potassium ferrocyanide (K₄[Fe(CN)₆]) under alkaline conditions is indicative of antioxidant activity. As ferricyanide and ferrocyanide exhibit differential absorbance at 700 nm, this reaction can be followed spectrophotometrically. As illustrated in Figure 4D, the mean absorbance of samples containing CPs at time 0 was 0.51, and it increased to 0.72 when the CPs-GOS conjugate was reacted for 8 h. Together, these radical scavenging assays demonstrated the antioxidant property of the CPs-GOS grafts due to the optimization of their structure and the introduction of active groups during the Maillard reaction.

**Figure 4:**
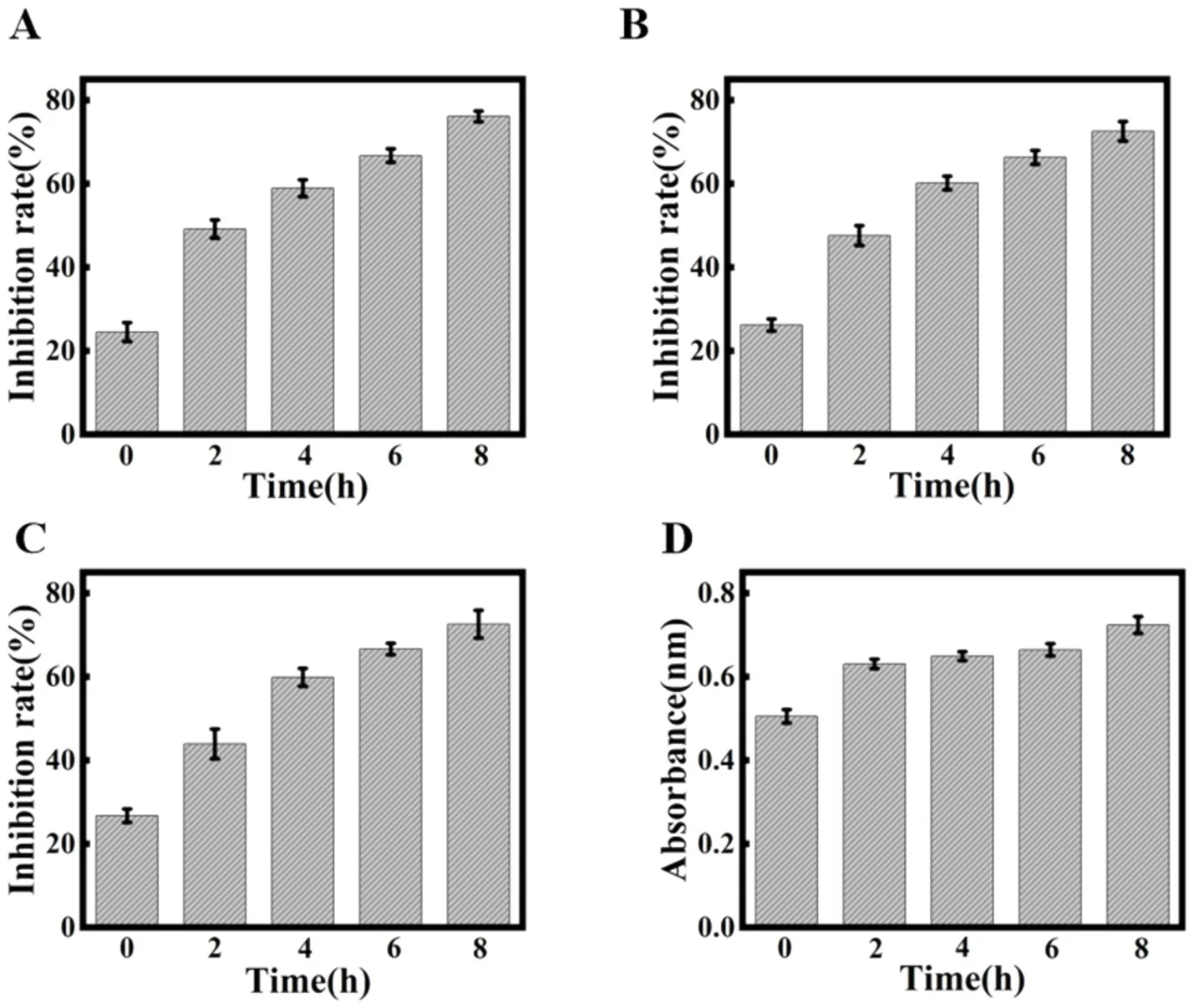
*In vitro* analyses of free radical scavenging activity. Free radical scavenging activities of CPs-GOS processed with different reaction times were assessed by **(A)** DPPH radical scavenging assay, **(B)** ABTS+ radical scavenging assay, **(C)** OH- free radical scavenging assay, and **(D)** potassium ferrocyanide reduction assay. Data presented as mean ± SD, *n* = 3.

### 3.5 CPs-GOS has minimal effects on cell viability

We initiated our study of interactions of CPs-GOS with living cells by testing the effects of incubation of LO2 cells with CPs-GOS on cell viability, as measured with the MTT assay. The LO2 cells were treated for 12 h with equal amounts of CPs-GOS produced with different reaction times (Figure 5A). We found that the viability of the cells was decreased slightly by incubation with the nanoparticles. The strongest decrease in viability was observed with CPs-GOS produced with a reaction time of 2 h, but additional reaction time was associated with lower losses of viability. After incubation for 12 h with CPs-GOS produced with an 8 h reaction time, the cell viability remained above 90%. We were excited to find that increasing the Maillard reaction time led to decreased cytotoxicity of the resulting CPs-GOS nanoparticle.

**Figure 5:**
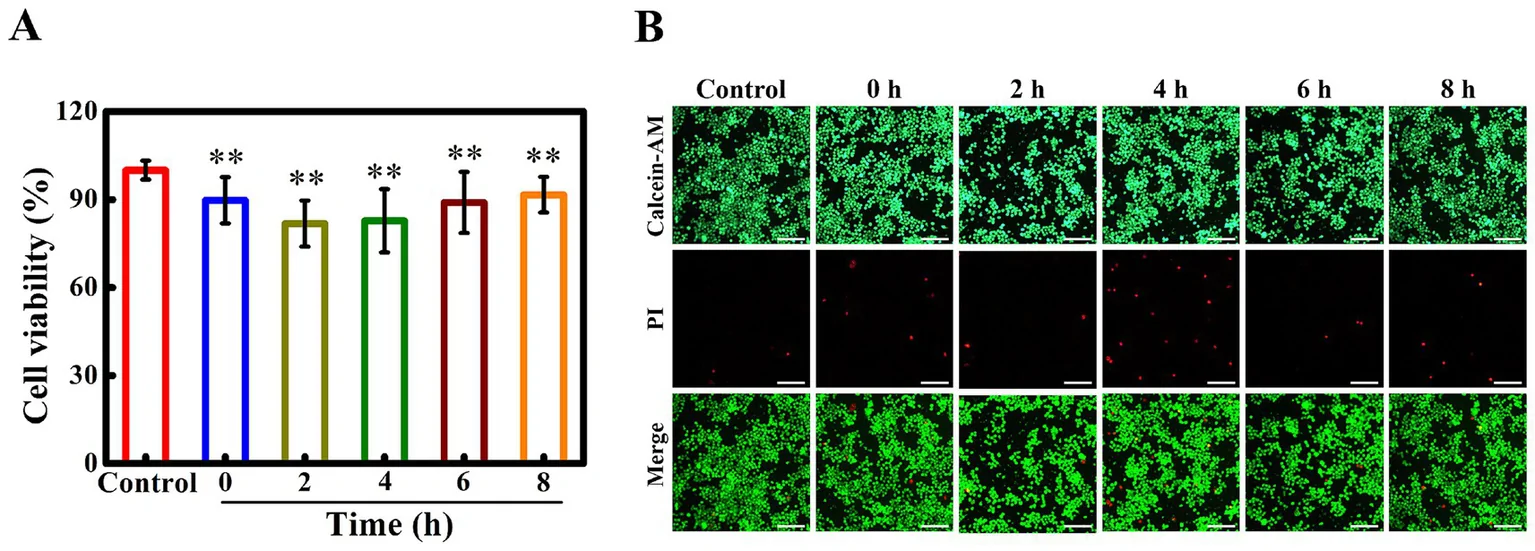
Impact of CP-GOP nanoparticles on cell viability. **(A)** MTT assay. Data presented as mean ± SD, *n* = 6. **p < 0.01 vs. vehicle control. **(B)** Representative Calcein-AM/PI images (green: live cells; red: dead cells). Scale bar = 100 μm.

Calcein-AM/PI staining was also employed as a measure of nanoparticle cytotoxicity in the LO2 cell model. As illustrated in Figure 5B, the cells in all treatment groups showed mainly green fluorescence (live cells) with few red fluorescence (dead cells), and these fluorescence patterns closely resembled those observed in the control group. Thus, these provide compelling evidence of the favorable safety profile of the CPs-GOS nanoparticles.

### 3.6 CPs-GOS alleviates ethanol-induced cytotoxicity

The safeguarding effect of CPs-GOS on ethanol-induced cellular injury was assessed in a LO2 cell model. In Figure 6A, ethanol treatment dramatically shortened cell viability to 48.87% relative to the control treatment. Remarkably, we observed a dose-dependent protective effect of CPs-GOS (8 h reaction time) in ethanol-treated cells. Specifically, the low, medium, and high doses of CPs-GOS rescued viability to 55.29%, 69.57%, and 75.18%, respectively, compared to that of control cells. These findings can be ascribed to the increase in bioavailability.

**Figure 6:**
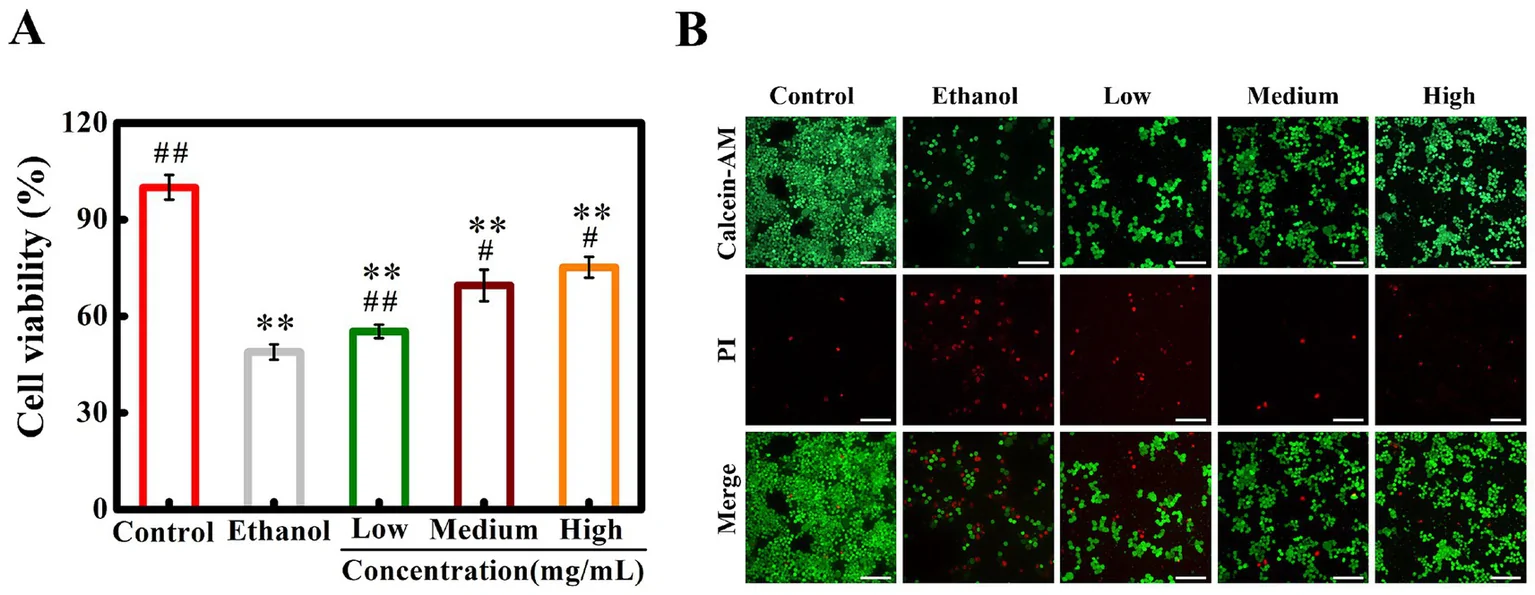
Protective effect of CPs-GOS against alcohol-induced toxicity. **(A)** MTT assay. The values were presented as mean ± SD (*n* = 6). **p < 0.01 vs. control (no ethanol, no CPs-GOS); #*p* < 0.05, ##*p* < 0.01 vs. ethanol alone. **(B)** Representative Calcein-AM/PI fluorescence images (green: live cells, red: dead cells). Scale bar = 100 μm.

The results of fluorescence staining assays (Figure 6B) were the same as the results of the MTT assays. While control cells showed minimal red signal, ethanol-treated cells showed intense red fluorescence, demonstrating the toxicity of ethanol in this system. In ethanol-injured cells treated with CPs-GOS, there was a reduction in red fluorescence signal, with the cells receiving the highest concentration showing the most notable enhancement, aligning with the results from the MTT assay. Collectively, these results demonstrate that CPs-GOS nanoparticles exhibit significant efficacy in protecting hepatocytes from ethanol-induced injury, underscoring their promise as a beneficial dietary supplement for preventing and managing ALD.

### 3.7 CPs-GOS nanoparticles are internalized by cultured cells

A progressive accumulation of the nanoparticles into cells would be essential to its bioactivity. To investigate internalization, we generated FITC-labeled CPs-GOS nano-particles and used them in fluorescence microscopy and flow cytometry assays (42). As illustrated in Figures 7A, B, CPs-GOS were internalized into LO2 cells in a time-dependent manner, with fluorescence intensity increasing from 2 to 4 h. The microscopic findings were corroborated by our flow cytometry analysis, which confirmed that the amount of FITC@CPs-GOS taken up by the LO2 cells increased with time.

**Figure 7:**
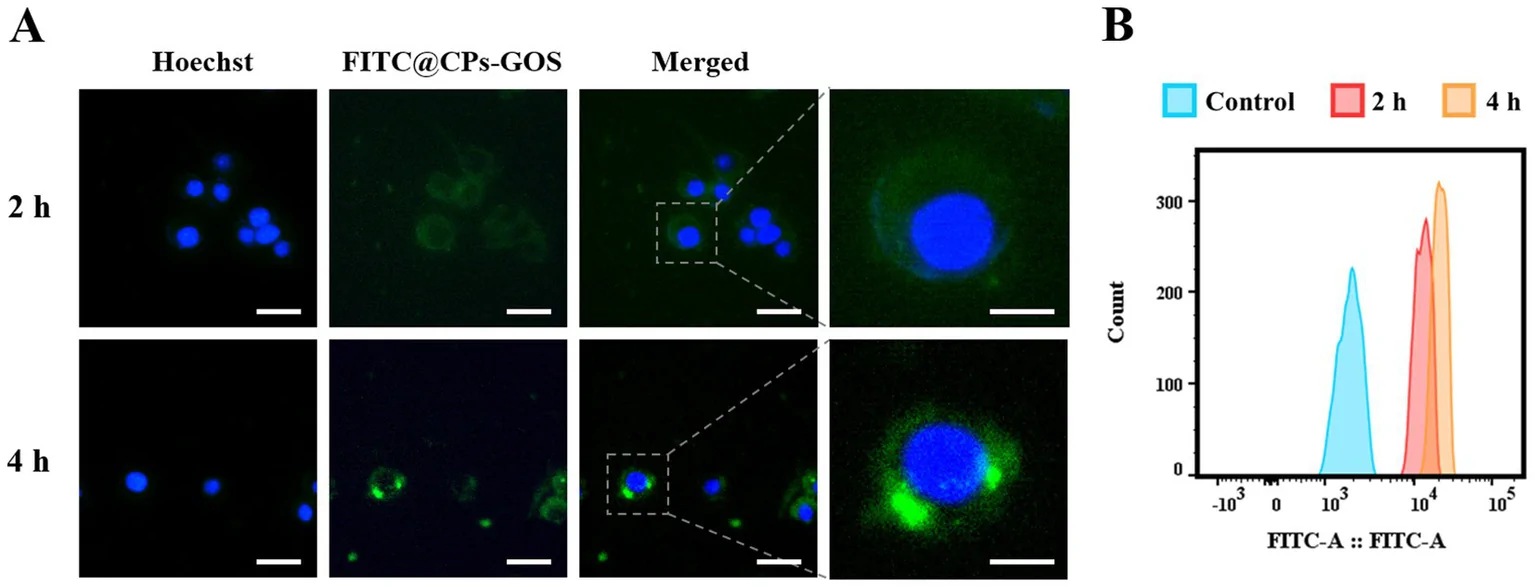
Cellular uptake of CPs-GOS. **(A)** Representative fluorescence microscopy images of LO2 cells treated with FITC-labeled CP-GOS (green) and counterstained with Hoechst 33342 (blue). Scale bar = 20 μm (Normal) and 5 μm (Enlarged). **(B)** Flow cytometry analysis of LO2 cells incubated with FITC-labeled CPs-GOS.

The uptake is likely mediated by galactosyl moieties on the nanoparticle. These chemical groups would be specifically recognized by ASGPR, which is highly expressed on hepatocyte membranes (25). Supplementary Figure S1 showed the ability to be specifically recognized by LO2 cells, which is consistent with the mechanism of galactose-targeted delivery reported by Wang et al. (43). The efficient internalization of CP-GOS set a foundation for subsequent experiments designed to investigate the hepatoprotection bioactivity of the nanoparticles.

### 3.8 CPs-GOS alleviates the ethanol-induced production of intracellular ROS

Reactive oxygen species (ROS) play a central role in ethanol-mediated cell damage. Following ethanol exposure, intracellular ROS levels rise abnormally, disrupting the antioxidant system. This oxidative insult promotes widespread damage, including oxidative degradation of proteins and lipids, DNA strand breaks, and loss of mitochondrial membrane potential, collectively progressing to cell death (44).

Here, we employed an ROS-sensitive fluorescent probe to quantitatively and qualitatively measure intracellular ROS levels. As illustrated in Figures 8A,B, ethanol exposure induced a pronounced rise in ROS, as indicated by increased fluorescence intensity, whereas treatment with CPs-GOS nanoparticles reduced the ethanol-enhanced ROS level in a concentration-dependent manner. This result indicates that CPs-GOS nanoparticles mitigate ethanol-triggered oxidative damage by scavenging excess ROS.

**Figure 8:**
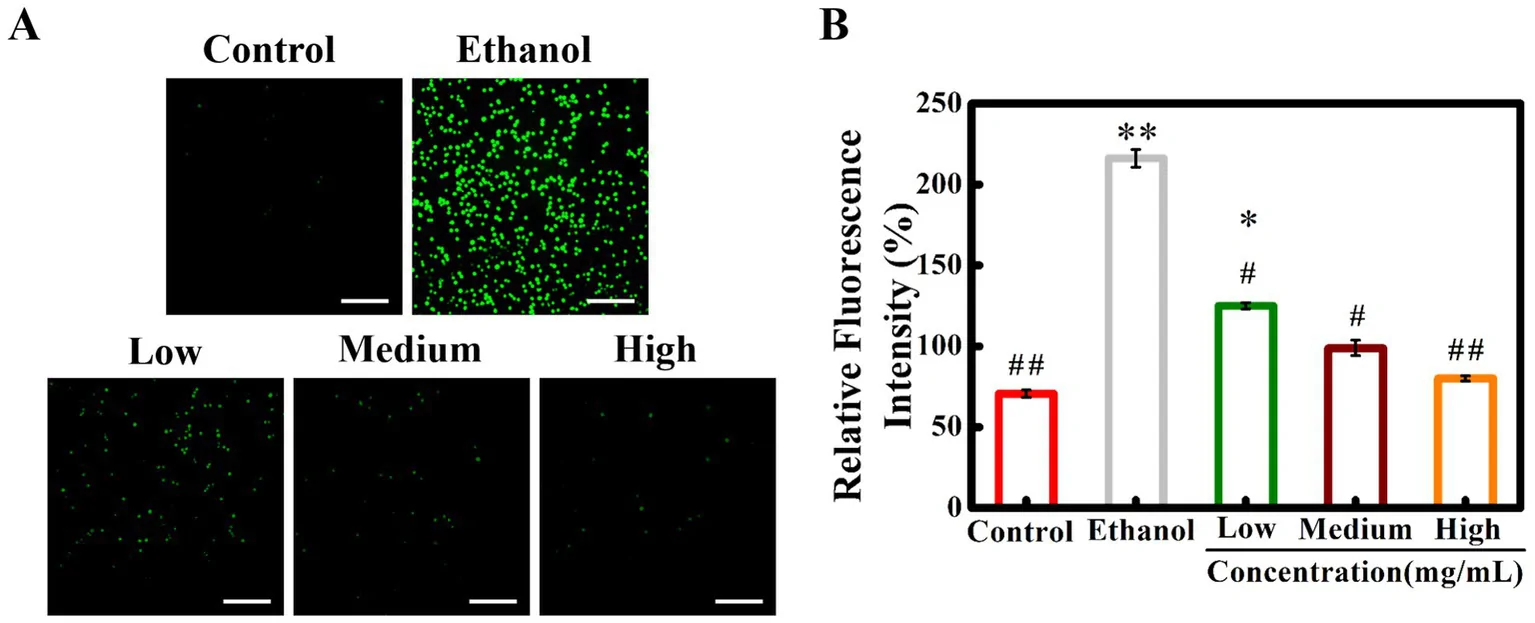
Mitigation of ethanol-induced ROS generation by CP-GOS (A) Representative fluorescence microscopy images. (control) and after treatment with (ethanol), CP-GOS nanoparticles (concentration: low, medium, and high). Scale bar = 100 μm **(B)** Quantification of fluorescence intensity in images as shown in **(A)**. Data presented as mean ± SD (*n* = 3). *p < 0.05, **p < 0.01 vs. untreated control cells; #*p* < 0.05, ##*p* < 0.01 vs. ethanol-treated cells without CP-GOS.

### 3.9 Preliminary investigation of biocompatibility

We found that CPs-GOS had only a modest impact on the viability of cultured LO2 cells (Figure 5). In order to further investigate the biocompatibility of the nanoparticles, we determined their hemolytic activity against blood, using a previously reported method (45). As illustrated in Figures 9A, B, CPs-GOS nanoparticles induced no detectable hemolysis when incubated with 0.25 mg/mL for 3 h with red blood cells. Quantitative analyses consistently showed low hemolysis ratios (0.06–0.14%), indicating that CPs-GOS do not damage red blood cells under the tested conditions. Collectively, these results confirm its hemocompatibility and suggest that it will exhibit an overall favorable safety profile during *in vivo* studies. Subsequent research will focus on using live animal models to determine the biodistribution of fluorescently labeled nanoparticles and to perform further validation of the safety profile of the formulation following oral administration.

**Figure 9:**
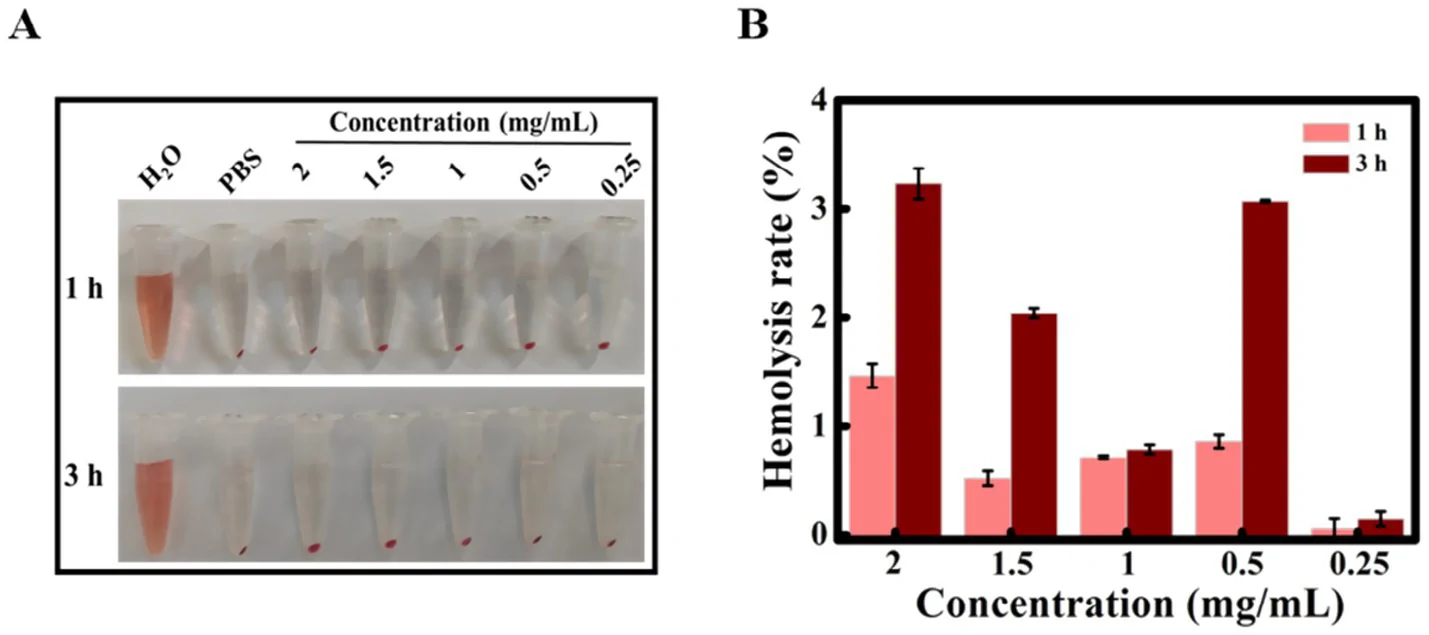
Hemolytic activity of CPs-GOS. *In vitro* hemolysis tests of CPs-GOS on **(A)** Hemolytic images at different concentrations and different time points; **(B)** Hemolytic rate.

## 4 Conclusion

Hepatocyte-targeting nanoparticles were effectively developed by glycosylating CPs with GOS. The resulting CPs-GOS nanoparticles displayed a spherical morphology, enhanced stability, and pronounced antioxidant capacity. Through ASGPR-mediated recognition of the galactosyl moiety, they demonstrated efficient cellular uptake and retention in LO2 cells. Functionally, the nanoparticles significantly mitigated ethanol-induced cytotoxicity and intracellular ROS overproduction, providing substantial protection against ethanol-induced cellular injury. Preliminary biosafety assessments confirmed negligible *in vitro* toxicity. Collectively, this work establishes GOS-glycosylated CPs as a promising targeted delivery system for alleviating alcohol-related cellular injury, with potential applicability for conveying other hydrophobic bioactive. While currently validated *in vitro*, upcoming research will concentrate on *in vivo* liver-specific targeting and therapeutic efficacy in models of alcoholic liver disease.

## Data Availability

The original contributions presented in the study are included in the article/Supplementary material, further inquiries can be directed to the corresponding author.
