# Supplementary material for: A hepatocyte targeted corn peptide—galactooligosaccharide nanoparticle for the alleviation of ethanol-induced damage in cells
Source: Front Nutr. 2026 Oct 27;13:1987391. doi: 10.3389/fnut.2026.1987391 (PMC13747776; doi:10.3389/fnut.2026.1987391)
Supplement: Supplementary file 1 [file Supplementary_file_1.doc]

A hepatocyte targeted corn peptide - galactooligosaccharide nanoparticle for the alleviation of ethanol-induced damage in cells

Figure S1. Fluorescence microscopy images of LO2 and Hela cells incubated with FITC@ CPs-GOS for 4 h (excitation wavelength: 445–490 nm).
